# Association of nutrient supplement use in older adults with chronic diseases: a cross-sectional study

**DOI:** 10.3389/fnut.2026.1727791

**Published:** 2026-02-11

**Authors:** Hua Tian, Jie Chen

**Affiliations:** 1 College of Tea and Food Science, Xinyang Normal University, Xinyang, China; 2 School of Marxism, Xinyang Normal University, Xinyang, China

**Keywords:** calcium, chronic diseases, female, nutrient supplements, vitamins

## Abstract

**Introduction:**

With advancing age, physiological and social changes increase the risk of malnutrition among older adults. Dietary supplements are a prevalent strategy for maintaining nutritional balance, enhancing health, and preventing chronic diseases.

**Methods:**

This study analyzed data from 9,986 participants aged 65 and older, drawn from the 2018 Chinese Longitudinal Healthy Longevity Survey (CLHLS), to compare supplement users and non-users. The data were analyzed using Mann–Whitney U test and Chi-square test.

**Results:**

The results revealed that only 11.91% older participants used nutrient supplements, and Calcium was the most used nutrient supplements. The typical user was women without spouses living with family, reporting a moderate lifestyle, high life satisfaction, and good health. There were potential associations between nutrient supplement use with chronic diseases, e.g., hypertension, heart disease, stroke and cerebrovascular disease, diabetes, and pneumonia or bronchitis.

**Conclusion:**

These findings suggest that nutrient supplement strategies should be personalized based on an individual’s chronic diseases. This research provides a crucial evidence base for health professionals to develop targeted interventions aimed at preventing the onset and deterioration of common chronic diseases in older adults.

## 1 Introduction

Dietary supplements are a prevalent strategy for maintaining nutritional balance, enhancing health, and preventing chronic diseases (1, 2). EU and US laws stipulate that nutrient supplements refer to concentrated sources of vitamins, minerals, or other substances with nutritional or physiological effects, such as amino acids, essential fatty acids, probiotics, plant and herbal extracts (3), present in small unit doses, aim to supple regular diets. Their composition, manufacturing, and safety are strictly regulated by extensive legislation at the EU and national levels (4). Nutrient supplements, despite their route of administration and appearance like drugs, have been classified as food rather than medicine (5, 6).

The relationship between nutrient supplements and chronic diseases is a core issue in modern public health and nutrition research. Nutrient supplements have the most significant effect on populations with nutritional deficiencies. Dietary surveys reveal that intakes of most vitamins and minerals fall below recommended levels, with older adults especially prone to deficiencies in key nutrients such as vitamin D and iron (7, 8). This vulnerability is linked to physiological and social changes associated with aging, which increase the risk of nutritional deficiencies (9). For instance, inadequate dietary protein can lead to a decline in lean body mass (LBM) and muscle weakness (10). Conversely, combining dietary protein supplementation with exercise is highly effective in mitigating age-related loss of muscle mass and leg strength (11). Ultimately, adequate nutritional status is integral to healthy aging, positively influencing the process, improving quality of life, alleviating chronic diseases, and delaying mortality (12, 13).

According to the 7th National Population Census of China, the population of older people aged 65 and above is approximately 191 million, accounting for 13.50% of the total population in the country (14). Due to aging, the appetite and food intake of older people decreases, resulting in reduced nutrition intake through diet (15). Older people are more prone to malnutrition, including nutrient deficiencies (16). The 2022 China Food and Nutrition Development Report showed that residents had imbalanced diets, and overweight and obesity among residents was constantly prominent. The prevalence and incidence of chronic diseases were still on the rise, which mainly manifested in the coexistence of overnutrition caused by excessive energy intake and nutritional deficiency caused by insufficient intake of vitamin A, calcium, iron and other micronutrients, and the high intake of oil, salt and sugar (17).

So far, there is no national representative report on Chinese older adults’ use characteristics of nutrient supplements and its association with chronic diseases. This study is the first to explore the use characteristics of nutrient supplements among older people in China based on the results of the Chinese Longitudinal Health Longevity Survey in 2018 (CLHLS-2018), and to explore the association between nutrient supplementation in older people and chronic diseases.

## 2 Methods

### 2.1 Study design

A cross-sectional descriptive and non-experimental association study was employed that relies on quantitative research methods. All participants were derived from the Chinese Longitudinal Healthy Longevity Survey in 2018 (CLHLS-2018). The CLHLS-2018 was based on face-to-face interview surveys of 15,874 participants aged 65 years and older from more than 500 investigation places in 22 provinces in China, who were selected randomly to participate in questionnaire responses. The first baseline survey conducted in 1998. Subsequently, nine surveys were conducted in 27 provinces, municipalities, and autonomous regions from 1998 to 2023. The goals of CLHLS are to discover the social, behavioral, environmental, and bio-medical factors and interactions that may influence healthy longevity and family happiness, as well as to provide data for academic research, and information for health and healthy aging policy analysis.

### 2.2 Participants

**Figure 1:**
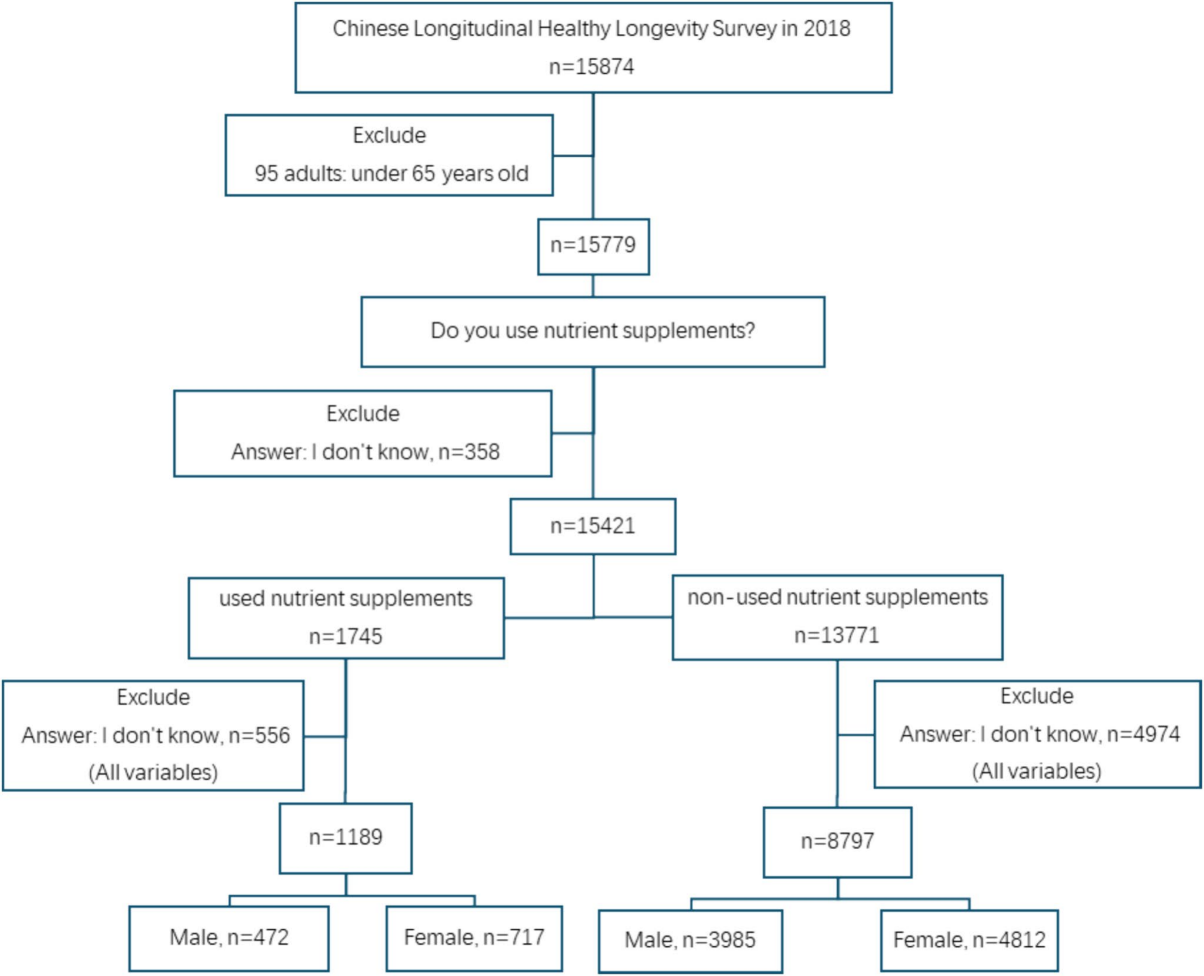
Study flowchart.

In this study, all participants were derived from CLHLS in 2018, aged 65 and above, and answered all questionnaire questions. If the answer was incomplete or there was no answer, the respondent should be deleted. Finally, 9,986 older adults (response rate: 62.91%) were selected as valid participants. The 9,986 participants were divided in two groups: used (male: 472, female: 717) and non-used nutrient supplements (male: 3,985, female: 4,812) to analyze Chinese older adults’ use characteristics of nutrient supplements and the association between nutrient supplement usage and chronic diseases (Figure 1).

### 2.3 Data analysis

Statistical analysis was performed using the SPSS v20 (SPSS v20, IBM Corp.: Chicago). The Mann- Whitney U test and Chi-square test were used for significant differences between continuous variables and categorical variables, respectively.

## 3 Results

### 3.1 Characteristics of participants

Table 1 showed the characteristics of 9,986 participants. The mean age was 84.23, and standard deviation was 11.68. Among them, 1,189 participants used nutrient supplements, and 8,797 participants non-used. The mean age and standard deviation of the former participants were 84.21 and 11.25, while the latter were 84.23 and 11.74.

**Table 1:** Characteristics of participants.

Characteristics	Total (*n* = 9,986)		Use (*n* = 1,189)		Non-use (*n* = 8,797)		*P*
	Frequency	%	Frequency	%	Frequency	%	
Gender							<0.001
Male	4,457	44.63	472	39.70	3,985	45.30	
Female	5,529	55.37	717	60.30	4,812	54.70	
Residence							<0.001
City	2,449	24.52	429	36.08	2,020	22.96	
Town	3,317	33.22	391	32.88	2,926	33.26	
Rural	4,220	42.26	369	31.03	3,851	43.78	
Age							0.964
Average	84.23 ± 11.68		84.21 ± 11.25		84.23 ± 11.74		
Older care types							0.001
Family	8,088	80.99	968	81.41	7,120	80.94	
Alone	1,576	15.78	164	13.79	1,412	16.05	
Care institutions	322	3.22	57	4.79	265	3.01	
Self-reported life satisfaction							<0.001
Excellent	2,388	23.91	385	32.38	2,003	22.77	
Good	4,670	46.77	545	45.84	4,125	46.89	
Neutral	2,624	26.28	235	19.76	2,389	27.16	
Poor	263	2.63	21	1.77	242	2.75	
Very poor	41	0.41	3	0.25	38	0.43	
Self-reported health							0.052
Excellent	1,223	12.25	165	13.88	1,058	12.03	
Good	3,561	35.66	428	36.00	3,133	35.61	
Neutral	3,843	38.48	423	35.58	3,420	38.88	
Poor	1,247	12.49	164	13.79	1,083	12.31	
Very poor	112	1.12	9	0.76	103	1.17	
Marriage							0.938
Unmarried	4,257	42.63	506	42.56	3,751	42.64	
Married	164	1.64	21	1.77	143	1.63	
No-spouse	5,565	55.73	662	55.68	4,903	55.73	
Living in affluence							<0.001
Excellent	274	2.74	49	4.12	225	2.56	
Good	1,748	17.50	315	26.49	1,433	16.29	
Neutral	7,002	70.12	753	63.33	6,249	71.04	
Poor	838	8.39	58	4.88	780	8.87	
Very poor	124	1.24	14	1.18	110	1.25	
Major chronic diseases							
Hypertension	4,204	42.10	559	47.01	3,645	41.43	<0.001
Heart diseases	1,692	16.94	291	24.47	1,401	15.93	<0.001
Stroke and cerebrovascular diseases	1,070	10.72	178	14.97	892	10.14	<0.001
Diabetes	1,022	10.23	166	13.96	856	9.73	<0.001
Pneumonia and bronchitis	1,006	10.07	141	11.86	865	9.83	0.031

*p* were calculated using the chi-square test and Mann- Whitney U test. The comparison between categorical variables was conducted using chi-square test, while the comparison between continuous variables was conducted using Mann- Whitney U test.

As for nutrient supplement users (*n* = 1,189), 60.30% were female and 54.16% divorced. Older participants from city, town, and rural each accounted for one-third. 81.41% lived with their families and 63.33% lived a moderate life with self-reported good health and life satisfaction. In summary, a significant feature of using nutrient supplements was that they were female without spouse, residing in cities, living in a neutral affluence life with their families and with good self-reported life satisfaction and health.

### 3.2 Nutrient supplements use among Chinese older people

Among 9,986 older participants in this study, only 11.91% (*n* = 1,189) used nutrient supplements. Seven commonly used nutrient supplements were counted, e.g., calcium, protein, multi-vitamins, vitamin A and D, iron, Zinc, DHA. As shown in Table 2, the most used nutrient supplement for older people was calcium (66.78%), followed by protein (20.35%), and multi-vitamins (18.84%). A total of 59.13% of older people had used nutrient supplements in the past 24 h. Subsequently, the types of nutrient supplements used were also statistically analyzed, limited to the above seven nutrient supplements. Among those who used nutrient supplements, 61.98% (*n* = 737) participants used one nutrient supplement, followed by 14.05% (*n* = 167) two nutrient supplements.

**Table 2:** Nutrient supplements usage of older adults in China (*n* = 1,189).

Nutrient supplements	*n*	%
Calcium	794	66.78
Protein	242	20.35
Multi-vitamins	224	18.84
Vitamin A and D	171	14.38
Iron	77	6.48
Zinc	69	5.80
DHA	47	3.95
Nutrient supplements used in the past 24 h	703	59.13

### 3.3 Characteristics of nutrient supplements users

**Figure 2:**
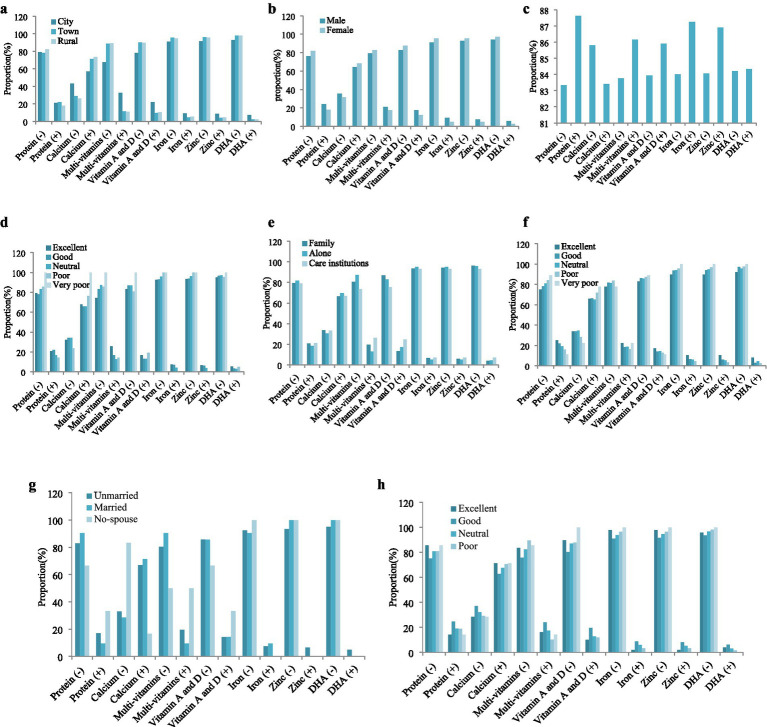
Characteristics of different nutrient supplements users (*n* = 1,189). Symbol “-” represented non-using nutrient supplements, and “+” represented the use of nutrient supplements. **(a)** Gender; **(b)** residence; **(c)** age; **(d)** self-reported life satisfaction; **(e)** older care types; **(f)** self-reported health; **(g)** marriage; **(h)** living in affluence.

As shown in Figure 2 and Appendix 1, the chi-square test found significant gender (*p* < 0.001) differences, age differences (*p* < 0.001), and marriage differences (*p* = 0.012) between protein supplement users and non-users. Using the same method, significant differences were found between residence (*p* < 0.001), and age (*p* = 0.001) with calcium nutrient supplement users and non-users, also residence (*p* < 0.001), age (*p* = 0.004), older care types (*p* = 0.045), self-reported life satisfaction (*p* = 0.001), living in affluence (*p* = 0.045) with multi-vitamins supplement users and non-users. Overall, there were significant differences in the use of nutrient supplements except for protein among older people in different residential areas, as well as significant age differences in the use of nutrient supplements except for DHA. It can be seen that residence and age were important factors for the use of nutritional supplements by the older people.

Therefore, Pearson chi-square multiple comparisons were conducted on the residence and Bonferroni correction was used to analyze the differences between pairwise combinations. As shown in Table 3, there were significant differences in the use of six other nutrient supplements (*p* < 0.01) between cities and towns, except for protein (*p* > 0.05). There were significant differences in the use of Calcium (*p* < 0.001), multi vitamins (*p* < 0.001), Vitamin A and D (*p* < 0.001) and DHA (*p* < 0.001) nutrient supplements between cities and rural areas. Additional, there was no significant difference in the use of six nutrient supplements between older people in towns and rural areas (*p* > 0.05).

**Table 3:** Pearson chi-square test pairwise comparison.

Characteristics	*n*	Calcium (%)			Multi-vitamins (%)			Vitamin A and D (%)			Iron (%)			Zinc (%)			DHA (%)		
		-	+	*P*	-	+	*P*	-	+	*P*	-	+	*P*	-	+	*P*	-	+	*P*
Residence				<0.001			<0.001			<0.001			0.013			0.014			<0.001
City	429	43.12	56.88		67.37	32.63		78.09	21.91		90.91	9.09		91.61	8.39		92.77	7.23	
Town	391	28.90	71.10		88.75	11.25		90.28	9.72		95.40	4.60		95.91	4.09		97.95	2.05	
				<0.001			<0.001			<0.001			0.057			0.033			<0.001
City	429	43.12	56.88		67.37	32.63		78.09	21.91		90.91	9.09		91.61	8.39		92.77	7.23	
Rural	369	26.29	73.71		89.16	10.84		89.43	10.57		94.58	5.42		95.39	4.61		97.83	2.17	
				0.465			0.908			0.720			0.622			0.859			0.907
Town	391	28.90	71.10		88.75	11.25		90.28	9.72		95.40	4.60		95.91	4.09		97.95	2.05	
Rural	369	26.29	73.71		89.16	10.84		89.43	10.57		94.58	5.42		95.39	4.61		97.83	2.17	

Pearson Chi-square multiple test was performed using Bonferroni to correct for significance level. In this table, the Bonferroni corrected significance level is 0.05/3 times = 0.0167.

### 3.4 Associations of nutrient supplements with chronic diseases

**Figure 3:**
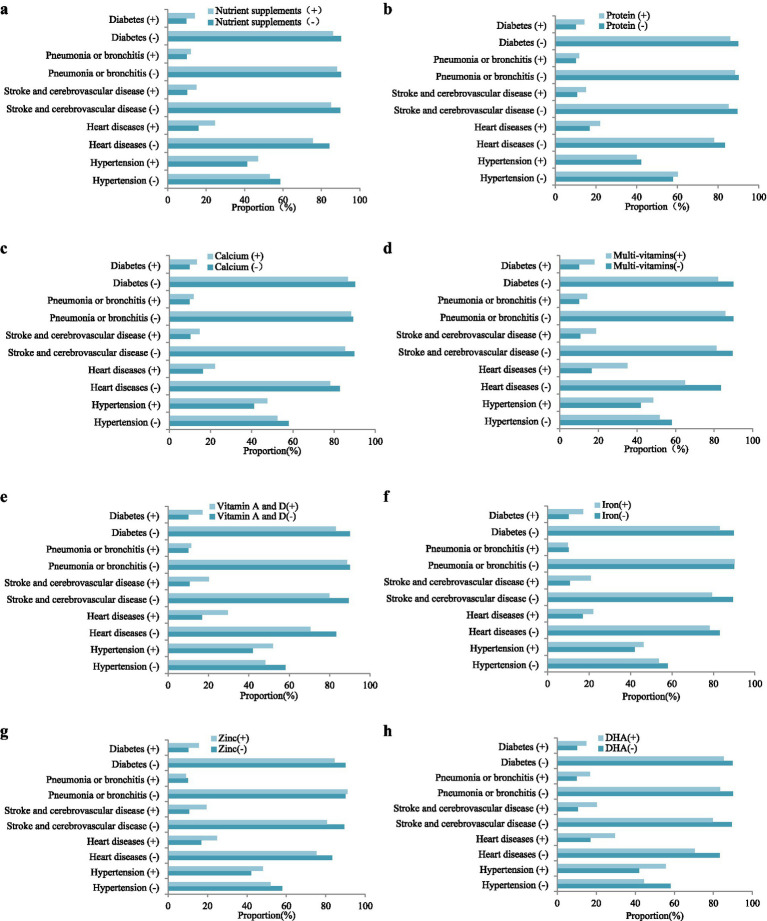
Associations of nutrient supplements with chronic diseases (*n* = 9,986). Symbol “-” represented non-using nutrient supplements or without disease, and “+” represented the use of nutrient supplements or with disease. **(a)** Nutrient supplements; **(b)** protein; **(c)** calcium; **(d)** multi-vitamins; **(e)** vitamin A and D; **(f)** iron; **(g)** zinc; **(h)** DHA.

Figure 3 and Appendix 2 showed associations of nutrient supplements usage and specific nutrient supplements with chronic diseases. Significant differences were found between nutrient supplements usage with hypertension (*p* < 0.001), heart diseases (*p* < 0.001), stroke and cerebrovascular diseases (*p* < 0.001), pneumonia and bronchitis (*p* = 0.031), and diabetes (*p* < 0.001), respectively. Specifically, for protein supplements, excluding hypertension (*p* = 0.491), pneumonia and bronchitis (*p* = 0.150), the other chronic diseases were significant associated with protein supplements (*p* < 0.05). Also, for calcium supplements, excluding pneumonia and bronchitis (*p* = 0.097), the other chronic diseases were significant associated with protein supplements (*p* < 0.05). For multi-vitamins, the common five chronic diseases were all significant associated with multi-vitamins supplement (*p* < 0.05). Vitamin A and D usage had significant associated with hypertension (*p* = 0.008), heart diseases (*p* < 0.001), stroke and cerebrovascular diseases (*p* < 0.001) and diabetes (*p* = 0.004). However, nutrient supplements usage of Iron, Zinc and DHA only had significant associated with stroke and cerebrovascular disease (*p* < 0.05), and had no significant associated with other four chronic diseases. Therefore, there was a significant difference (*p* < 0.05) in the incidence of various chronic diseases between the group using and non-using nutrient supplements, especially in the use of multi-vitamins, Calcium and Vitamin A and D.

## 4 Discussion

The study yielded three primary findings. First, only 11.91% older participants used nutrient supplements, and Calcium was the most used nutrient supplement for the older people. Second, female without spouse represented the largest user demographic. Third, there were potential associations in the incidence of chronic diseases between usage nutrient supplements group and non-usage group.

In the present study, the utilization of nutritional supplements among older adults was merely 11.91%, a figure starkly lower than the prevalence rates exceeding 50% recently documented in Western countries (18). This pronounced discrepancy likely reflects distinct cultural dietary attitudes, where traditional food sources may be prioritized over supplementation, alongside potential gaps in health literacy regarding preventive nutrition. The observed dominance of calcium, protein, and multivitamins aligns with current clinical guidelines aimed at mitigating age-related conditions, particularly osteoporosis and sarcopenia (19). However, the overall low uptake highlights a critical need for targeted public health education to bridge the gap between nutritional awareness and actual supplementation practices among the older population.

The nutrient supplements user was woman group without spouse co-residing with family, which corroborates recent findings indicating that women possess higher health awareness and are more proactive in preventive care compared to men (20). While divorce and widowed are often linked to adverse health outcomes, our findings suggest that for this specific demographic, increased care and support from co-residing family members act as a protective mechanism, facilitating positive health behaviors (21). Furthermore, the strong association between high life satisfaction and nutrient supplement use supports recent evidence linking subjective well-being to proactive health management in older adults (22). Despite these insights, the specific mechanisms linking widowhood, divorced, family dynamics, and supplementation in the Chinese context remain under-researched, warranting further longitudinal investigation.

Moreover, significant differences in the incidence of chronic diseases—such as hypertension, cardiovascular disease, stroke, diabetes, pneumonia, and bronchitis—were found between the nutritional supplement user group and the non-user group. This distinction was most pronounced in the consumption of multivitamins, calcium, and vitamins A and D. Research demonstrates that micronutrient deficiencies (notably of iron and vitamins A, C, D, and E) are more common in older adults and correlate with oxidative stress (23), mitochondrial dysfunction, and neurodegeneration. Such deficiencies may consequently contribute to the pathogenesis of Alzheimer’s (16) and Parkinson’s diseases (24, 25). Furthermore, consistent multivitamin supplementation can mitigate the incidence of age-related chronic conditions (26). A potential association has also been identified between digestive system tumors and the intake of protein, lycopene, and various micronutrients, specifically vitamins B1, B2, B12, D, E, and folate, along with minerals such as copper, calcium, iron, phosphorus, and zinc (27). Additional, nutrient intake significantly affects the incidence of gastric cancer (28).

Participants with poor health status exhibited the highest utilization rates of nutritional supplements, while those unaware of their health status were the least likely to use them (*p* < 0.05; (29)). This correlation emphasizes that while nutritional supplements have therapeutic potential, indiscriminate or excessive consumption can pose significant risks. Empirical evidence indicates that administering vitamins and minerals without clear medical indications neither reduces the risk of cardiovascular disease or cancer nor guarantees safety, potentially inducing adverse effects such as hypervitaminosis or drug-nutrient interactions (30). Thus, the disparity between usage and clinical outcomes underscores the critical need for a personalized, precision-nutrition approach to supplementation, ensuring that intake is tailored to individual metabolic profiles and health requirements (31).

This study has several limitations that should be acknowledged. First, the cross-sectional design precludes the determination of causality. Second, while the analysis included the full sample of 9,986 eligible participants, sensitivity and specificity calculations for the questionnaire itself were not performed. Finally, reliance on self-reported questionnaires means the results may be subject to recall or social desirability bias. Despite these limitations, the potential associations identified between nutritional supplement use and listed chronic diseases which provided a valuable reference for healthcare professionals seeking to develop nutritional strategies to prevent and manage common chronic conditions in older adults.

## 5 Conclusion

Among the 9,986 participants from the CLHLS-2018, 1,189 reported regular use of nutrient supplements. Calcium was the most frequently consumed nutrient supplement, followed by protein and multivitamins. The typical user profile was characterized as woman group without spouse, living with family members who self-reported moderate socioeconomic status, high life satisfaction, and good overall health. Residence and age were identified as significant determinants of nutrient supplement use among the older people. There were potential associations between the prevalence of chronic diseases with nutrient supplement use in older adults, particularly with the multivitamins, calcium, and vitamins A and D. Based on these findings, future research should focus on: (1) investigating barriers to uptake, specifically why overall usage remains low and predominantly calcium-focused, to determine if other critical micronutrients are being neglected; (2) exploring psychosocial drivers to develop targeted interventions for underrepresented groups; and (3) establishing causal pathways, utilizing longitudinal studies to validate whether these supplement associations can genuinely prevent the progression of chronic diseases or if they reflect reverse causation.

## Data Availability

The original contributions presented in the study are included in the article/Supplementary material, further inquiries can be directed to the corresponding author.

## References

[1] Jaisamrarn U Habana MA Damodaran P Tint MT Chuang HH Hunter DJ . Healthy aging in midlife and menopausal transition in Asia: nutrient synergy with dietary supplements. Climacteric. (2025) 3:1–15. doi: 10.1080/13697137.2025.2567689, 41182953

[2] Shi Y Li P Jiang CF Chen Y Ma Y Gupta N . Identification of accessible hepatic gene signatures for inter individual variations in nutrigenomic response to dietary supplementation of omega-3 fatty acids. Cells. (2021) 10:467. doi: 10.3390/cells10020467 33671567 PMC7926558

[3] Petkova-Gueorguieva ES Getov IN Ivanov KV Ivanova SD Gueorguiev SR Getova VI . Regulatory requirements for food supplements in the European Union and Bulgaria. Folia Med. (2019) 61:41–8. doi: 10.2478/folmed-2018-0032, 31237857

[4] Coppens P. The importance of food supplements for public health and well-being. World Rev Nutr Diet. (2020) 121:66–72. doi: 10.1159/000507524, 33502375

[5] Dominguez Diaz L Fernandez-Ruiz V Camara M. The frontier between nutrition and pharma: the international regulatory framework of functional foods, food supplements and nutraceuticals. Crit Rev Food Sci Nutr. (2020) 60:1738–46. doi: 10.1080/10408398.2019.1592107, 30924346

[6] Jain N Radhakrishnan A Kuppusamy G. Review on nutraceuticals: phase transition from preventive to protective care. J Complement Integr Med. (2022) 19:553–70. doi: 10.1515/jcim-2022-0026, 35436045

[7] MacDonell SO Miller JC Fleming EA Haszard JJ. Inadequate micronutrient intakes: exploring the vitamin and mineral intakes and food sources of New Zealand aged-care residents. J Hum Nutr Diet. (2025) 38:e70123. doi: 10.1111/jhn.70123, 40923637 PMC12418734

[8] Stahl-Gugger A de Godoi Rezen Costa Molino C Wieczorek M Chocano-Bedoya PO Abderhalden LA Schaer DJ . Prevalence and incidence of iron deficiency in European community-dwelling older adults: an observational analysis of the DO-HEALTH trial. Aging Clin Exp Res. (2022) 34:2205–15. doi: 10.1007/s40520-022-02093-0, 35304704 PMC9464157

[9] Kehoe L Walton J Flynn A. Nutritional challenges for older adults in Europe: current status and future directions. Proc Nutr Soc. (2019) 78:221–33. doi: 10.1017/S0029665118002744, 30696516

[10] Mohammadi S Ashtary-Larky D Alaghemand N Alnsour AF Shokouhifar S Borzabadi A. Effects of supplementation with milk proteins on body composition and anthropometric parameters: a systematic review and dose-response meta-analysis. Nutrients. (2025) 17:3877. doi: 10.3390/nu17243877, 41470822 PMC12736298

[11] Imaoka M Hida M Nakamura M Sakai K Anzai E Ichise T . The effect of exercise and soy protein intake on physical frailty score improvement in community-dwelling elderly: a randomized controlled trial. Exp Gerontol. (2026) 213:112995. doi: 10.1016/j.exger.2025.112995, 41386386

[12] Canamares-Orbis P Garcia-Rayado G Alfaro-Almajano E. Nutritional support in pancreatic diseases. Nutrients. (2022) 14:4570. doi: 10.3390/nu14214570, 36364832 PMC9656643

[13] Changaramkumarath G Abucha JM Wollel MM Somannagari N Kasagga A Sapkota A . Pharmacological interactions between nutritional supplements and prescription medications in older adults: a comprehensive review. Cureus. (2025) 17:e92363. doi: 10.7759/cureus.92363, 41103884 PMC12526737

[14] National Bureau of Statistics. (2021) Communique of the Seventh National Population Census. Available online at: http://www.gov.cn/guoqing/2021-05/13/content_5606149.htm

[15] Vural Z Avery A Kalogiros DI Coneyworth LJ Welham SJM. Trace mineral intake and deficiencies in older adults living in the community and institutions: a systematic review. Nutrients. (2020) 12:1072. doi: 10.3390/nu12041072, 32294896 PMC7230219

[16] Li SY Sun WJ Zhang DF. Association of Zinc, Iron, copper, and selenium intakes with low cognitive performance in older adults: a cross-sectional Study from National Health and nutrition examination survey (NHANES). J Alzheimer's Dis. (2019) 72:1145–57. doi: 10.3233/JAD-190263, 31683474

[17] Institute of Food and Nutrition Development of the Ministry of Agriculture and Rural Affairs. China food and nutrition development report in 2022. Beijing: Agricultural Science and Technology Press (2023).

[18] Zhao L Zhang Y Liu J Hebert JR Giovannucci E Zhang X . Trends in dietary supplement use among U.S. adults between 2011 and 2023. Eur J Nutr. (2025) 64:304. doi: 10.1007/s00394-025-03825-4, 41128907

[19] Coelho-Junior HJ Calvani R Azzolino D Picca A Tosato M Landi F . Protein intake and sarcopenia in older adults: a systematic review and meta-analysis. Int J Environ Res Public Health. (2022) 19:8718. doi: 10.3390/ijerph19148718, 35886571 PMC9320473

[20] Garg V Lin J Gadzhieva-Moore A Yee SM Dhiman A Kumari R . The role of multivitamin and mineral supplements in supporting health and well-being: a retrospective cross-sectional study in Taiwan. J Health Popul Nutr. (2025) 44:423. doi: 10.1186/s41043-025-01164-y, 41437132 PMC12729314

[21] Ye M Hu J. From disconnection to well-being: a longitudinal study on digital access as a social determinant of health for older adults in China. J Gerontol B Psychol Sci Soc Sci. (2026) 81:gbaf211. doi: 10.1093/geronb/gbaf211, 41138162

[22] Li Z Li D Li Z. Digital literacy, life satisfaction, social capital, and multidimensional health behaviors among middle-aged and older adults in rural China: a nationwide cross-sectional study. Digit health. (2025) 11:20552076251374114. doi: 10.1177/20552076251374114, 41018519 PMC12464424

[23] Rowe S Carr AC. Global vitamin C status and prevalence of deficiency: a cause for concern? Nutrients. (2020) 12:2008. doi: 10.3390/nu12072008, 32640674 PMC7400810

[24] Kumar RR Singh L Thakur A Singh S Kumar B. Role of vitamins in neurodegenerative diseases: a review. CNS Neurol Disord Drug Targets. (2022) 21:766–73. doi: 10.2174/1871527320666211119122150, 34802410

[25] Wylenzek F Buhling KJ Laakmann E. A systematic review on the impact of nutrition and possible supplementation on the deficiency of vitamin complexes, iron, omega-3-fatty acids, and lycopene in relation to increased morbidity in women after menopause. Arch Gynecol Obstet. (2024) 310:2235–45. doi: 10.1007/s00404-024-07555-6, 38935105 PMC11393286

[26] Soos R Bako C Gyebrovszki A Gordos M Csala D Adam Z . Nutritional habits of Hungarian older adults. Nutrients. (2024) 16:1203. doi: 10.3390/nu16081203, 38674893 PMC11053580

[27] Qin X Ge L Wu S Li W. Association of dietary intake with cancer of the digestive system: a cross-sectional study. Front Nutr. (2025) 12:1539401. doi: 10.3389/fnut.2025.1539401, 39911800 PMC11796475

[28] Kim YN Kim CY. Exploration of the relationship between gastric cancer and nutritional risk factors: insights from the Korea National Health Insurance Database. Front Nutr. (2025) 12:1538133. doi: 10.3389/fnut.2025.1538133, 40432962 PMC12106021

[29] Gong W Liu A Yao Y Ma Y Ding C Song C . Nutrient supplement use among the Chinese population: a cross-sectional study of the 2010-2012 China nutrition and health surveillance. Nutrients. (2018) 10:1733. doi: 10.3390/nu10111733, 30424493 PMC6266204

[30] Wierzejska RE. Dietary supplements-for whom? The current state of knowledge about the health effects of selected supplement use. Int J Environ Res Public Health. (2021) 18:8897. doi: 10.3390/ijerph18178897, 34501487 PMC8431076

[31] Khandelwal S Lane NE. Osteoporosis: review of etiology, mechanisms, and approach to Management in the Aging Population. Endocrinol Metab Clin N Am. (2023) 52:259–75. doi: 10.1016/j.ecl.2022.10.009, 36948779

