# Supplementary material for: Association of nutrient supplement use in older adults with chronic diseases: a cross-sectional study
Source: Front Nutr. 2026 Feb 11;13:1727791. doi: 10.3389/fnut.2026.1727791 (PMC12932216; doi:10.3389/fnut.2026.1727791)
Supplement: Supplementary file 1 [file Table_1.docx]

Appendix 1 Characteristics of different nutrient supplements users (n=1,189).

| Characteristics | n | Protein (%) | | | Calcium (%) | | | Multi-vitamins (%) | | | Vitamin A and D (%) | | | Iron (%) | | | Zinc (%) | | | DHA (%) | | |
| --- | --- | --- | --- | --- | --- | --- | --- | --- | --- | --- | --- | --- | --- | --- | --- | --- | --- | --- | --- | --- | --- | --- |
|  |  | - | + | *P* | - | + | *P* | - | + | *P* | - | + | *P* | - | + | *P* | - | + | *P* | - | + | *P* |
| Gender |  |  |  | < 0.001 |  |  | 0.159 |  |  | 0.127 |  |  | 0.018 |  |  | 0.004 |  |  | 0.058 |  |  | 0.014 |
| Male | 472 | 76.06 | 23.94 |  | 35.59 | 64.41 |  | 79.03 | 20.97 |  | 82.63 | 17.37 |  | 90.89 | 9.11 |  | 92.58 | 7.42 |  | 94.28 | 5.72 |  |
| Female | 717 | 82.01 | 17.99 |  | 31.66 | 68.34 |  | 82.57 | 17.43 |  | 87.59 | 12.41 |  | 95.26 | 4.74 |  | 95.26 | 4.74 |  | 97.21 | 2.79 |  |
| Residence |  |  |  | 0.343 |  |  | <0.001 |  |  | <0.001 |  |  | <0.001 |  |  | 0.020 |  |  | 0.016 |  |  | <0.001 |
| City | 429 | 79.02 | 20.98 |  | 43.12 | 56.88 |  | 67.37 | 32.63 |  | 78.09 | 21.91 |  | 90.91 | 9.09 |  | 91.61 | 8.39 |  | 92.77 | 7.23 |  |
| Town | 391 | 78.01 | 21.99 |  | 28.90 | 71.10 |  | 88.75 | 11.25 |  | 90.28 | 9.72 |  | 95.40 | 4.60 |  | 95.91 | 4.09 |  | 97.95 | 2.05 |  |
| Rural | 369 | 82.11 | 17.89 |  | 26.29 | 73.71 |  | 89.16 | 10.84 |  | 89.43 | 10.57 |  | 94.58 | 5.42 |  | 95.39 | 4.61 |  | 97.83 | 2.17 |  |
| Age |  |  |  | < 0.001 |  |  | 0.001 |  |  | 0.004 |  |  | 0.033 |  |  | 0.014 |  |  | 0.040 |  |  | 0.938 |
| Average |  | 83.34 | 87.62 |  | 85.82 | 83.41 |  | 83.76 | 86.16 |  | 83.93 | 85.91 |  | 84.00 | 87.25 |  | 84.05 | 11.24 |  | 84.21 | 11.26 |  |
| SD |  | 11.05 | 11.39 |  | 11.22 | 11.18 |  | 11.27 | 10.97 |  | 11.28 | 10.52 |  | 11.26 | 10.70 |  | 86.91 | 11.05 |  | 84.34 | 11.00 |  |
| Older care types |  |  |  | 0.777 |  |  | 0.725 |  |  | 0.045 |  |  | 0.036 |  |  | 0.667 |  |  | 0.809 |  |  | 0.451 |
| Family | 968 | 79.34 | 20.66 |  | 33.68 | 66.32 |  | 80.58 | 19.42 |  | 86.67 | 13.33 |  | 93.29 | 6.71 |  | 94.11 | 5.89 |  | 96.28 | 3.72 |  |
| Alone | 164 | 81.71 | 18.29 |  | 30.49 | 69.51 |  | 87.20 | 12.80 |  | 82.93 | 17.07 |  | 95.12 | 4.88 |  | 95.12 | 4.88 |  | 95.73 | 4.27 |  |
| Care institutions | 57 | 78.95 | 21.05 |  | 33.33 | 66.67 |  | 73.68 | 26.32 |  | 75.44 | 24.56 |  | 92.98 | 7.02 |  | 92.98 | 7.02 |  | 92.98 | 7.02 |  |
| Self-reported life satisfaction | | | | 0.371 |  |  | 0.595 |  |  | 0.001 |  |  | 0.505 |  |  | 0.342 |  |  | 0.409 |  |  | 0.623 |
| Excellent | 385 | 79.22 | 20.78 |  | 32.21 | 67.79 |  | 74.29 | 25.71 |  | 83.38 | 16.62 |  | 92.47 | 7.53 |  | 93.25 | 6.75 |  | 94.81 | 5.19 |  |
| Good | 545 | 77.98 | 22.02 |  | 34.13 | 65.87 |  | 83.12 | 16.88 |  | 86.79 | 13.21 |  | 93.03 | 6.97 |  | 93.76 | 6.24 |  | 96.51 | 3.49 |  |
| Neutral | 235 | 83.40 | 16.60 |  | 34.04 | 65.96 |  | 87.23 | 12.77 |  | 86.81 | 13.19 |  | 95.74 | 4.26 |  | 96.17 | 3.83 |  | 97.02 | 2.98 |  |
| Poor | 21 | 85.71 | 14.29 |  | 23.81 | 76.19 |  | 85.71 | 14.29 |  | 80.95 | 19.05 |  | 100 | 0.00 |  | 100 | 0 |  | 95.24 | 4.76 |  |
| Very poor | 3 | 100 | 0 |  | 0 | 100 |  | 100 | 0 |  | 100 | 0 |  | 100 | 0 |  | 100 | 0 |  | 100 | 0 |  |
| Self-reported health | | |  | 0.214 |  |  | 0.560 |  |  | 0.716 |  |  | 0.852 |  |  | 0.188 |  |  | 0.506 |  |  | 0.047 |
| Excellent | 165 | 75.15 | 24.85 |  | 33.94 | 66.06 |  | 77.58 | 22.42 |  | 83.03 | 16.97 |  | 89.70 | 10.30 |  | 89.70 | 10.30 |  | 92.12 | 7.88 |  |
| Good | 428 | 78.04 | 21.96 |  | 33.64 | 66.36 |  | 81.54 | 18.46 |  | 85.98 | 14.02 |  | 93.46 | 6.54 |  | 94.16 | 5.84 |  | 97.20 | 2.80 |  |
| Neutral | 423 | 81.09 | 18.91 |  | 34.75 | 65.25 |  | 81.32 | 18.68 |  | 85.58 | 14.42 |  | 94.09 | 5.91 |  | 94.80 | 5.20 |  | 95.74 | 4.26 |  |
| Poor | 164 | 84.15 | 15.85 |  | 28.05 | 71.95 |  | 83.54 | 16.46 |  | 87.20 | 12.80 |  | 95.73 | 4.27 |  | 96.95 | 3.05 |  | 97.56 | 2.44 |  |
| Very poor | 9 | 88.89 | 11.11 |  | 22.22 | 77.78 |  | 77.78 | 22.22 |  | 88.89 | 11.11 |  | 100 | 0 |  | 100 | 0 |  | 100 | 0 |  |
| Marriage |  |  |  | 0.880 |  |  | 0.212 |  |  | 0.163 |  |  | 0.951 |  |  | 0.760 |  |  | 0.787 |  |  | 0.772 |
| Unmarried | 506 | 97.04 | 2.96 |  | 87.75 | 12.25 |  | 92.89 | 7.11 |  | 96.44 | 3.56 |  | 98.02 | 1.98 |  | 98.22 | 1.78 |  | 98.62 | 1.38 |  |
| Married | 21 | 95.24 | 4.76 |  | 85.71 | 14.29 |  | 95.24 | 4.76 |  | 95.24 | 4.76 |  | 100 | 0 |  | 100 | 0 |  | 100 | 0 |  |
| No-spouse | 662 | 97.13 | 2.87 |  | 90.79 | 9.21 |  | 95.47 | 4.53 |  | 96.53 | 3.47 |  | 98.34 | 1.66 |  | 98.49 | 1.51 |  | 98.94 | 1.06 |  |
| Living in affluence |  |  |  | 0.201 |  |  | 0.226 |  |  | 0.045 |  |  | 0.018 |  |  | 0.157 |  |  | 0.164 |  |  | 0.123 |
| Excellent | 49 | 85.71 | 14.29 |  | 28.57 | 71.43 |  | 83.67 | 16.33 |  | 89.80 | 10.20 |  | 97.96 | 2.04 |  | 97.96 | 2.04 |  | 95.92 | 4.08 |  |
| Good | 315 | 75.24 | 24.76 |  | 37.14 | 62.86 |  | 75.87 | 24.13 |  | 80.32 | 19.68 |  | 91.11 | 8.89 |  | 91.75 | 8.25 |  | 93.65 | 6.35 |  |
| Neutral | 753 | 80.88 | 19.12 |  | 32.27 | 67.73 |  | 82.47 | 17.53 |  | 87.12 | 12.88 |  | 93.89 | 6.11 |  | 94.69 | 5.31 |  | 96.81 | 3.19 |  |
| Poor | 58 | 81.03 | 18.97 |  | 29.31 | 70.69 |  | 89.66 | 10.34 |  | 87.93 | 12.07 |  | 96.55 | 3.45 |  | 96.55 | 3.45 |  | 98.28 | 1.72 |  |
| Very poor | 14 | 85.71 | 14.29 |  | 28.57 | 71.43 |  | 85.71 | 14.29 |  | 100 | 0 |  | 100 | 0 |  | 100 | 0 |  | 100 | 0 |  |

Note. Symbol “-” represented non-using nutrient supplements, and “+” represented the use of nutrient supplements. The figure below the symbol represented the proportion of older people using or non-using specific nutrient supplements. P-values were calculated using the chi-square and t-tests.

Appendix 2 Associations of nutrient supplements with chronic diseases (n=9,986).

| Characteristics | n | Hypertension (%) | | | Heart diseases (%) | | | Stroke and cerebrovascular disease (%) | | | Pneumonia or bronchitis (%) | | | Diabetes (%) | | |
| --- | --- | --- | --- | --- | --- | --- | --- | --- | --- | --- | --- | --- | --- | --- | --- | --- |
|  |  | - | + | P | - | + | P | - | + | P | - | + | P | - | + | P |
| Nutrient supplements |  |  |  | <0.001 |  |  | <0.001 |  |  | <0.001 |  |  | 0.031 |  |  | <0.001 |
| - | 8797 | 58.57 | 41.43 |  | 84.07 | 15.93 |  | 89.86 | 10.14 |  | 90.17 | 9.83 |  | 90.27 | 9.73 |  |
| + | 1189 | 52.99 | 47.01 |  | 75.53 | 24.47 |  | 85.03 | 14.97 |  | 88.14 | 11.86 |  | 86.04 | 13.96 |  |
| Protein |  |  |  | 0.491 |  |  | 0.031 |  |  | 0.028 |  |  | 0.150 |  |  | 0.040 |
| - | 9718 | 57.84 | 42.16 |  | 83.20 | 16.80 |  | 89.40 | 10.60 |  | 90.00 | 10.00 |  | 89.87 | 10.13 |  |
| + | 268 | 60.07 | 39.93 |  | 77.99 | 22.01 |  | 85.07 | 14.93 |  | 88.31 | 11.69 |  | 85.82 | 14.18 |  |
| Calcium |  |  |  | 0.001 |  |  | <0.001 |  |  | <0.001 |  |  | 0.097 |  |  | 0.002 |
| - | 9124 | 57.85 | 41.18 |  | 82.72 | 16.30 |  | 89.78 | 10.22 |  | 89.20 | 9.82 |  | 90.18 | 9.82 |  |
| + | 862 | 52.44 | 47.56 |  | 77.96 | 22.04 |  | 85.38 | 14.62 |  | 88.28 | 11.72 |  | 86.66 | 13.34 |  |
| Multi-vitamins |  |  |  | 0.048 |  |  | <0.001 |  |  | <0.001 |  |  | 0.039 |  |  | <0.001 |
| - | 9746 | 58.05 | 41.95 |  | 83.50 | 16.50 |  | 89.48 | 10.52 |  | 90.03 | 9.97 |  | 89.95 | 10.05 |  |
| + | 240 | 51.67 | 48.33 |  | 65.00 | 35.00 |  | 81.25 | 18.75 |  | 85.83 | 14.17 |  | 82.08 | 17.92 |  |
| Vitamin A and D |  |  |  | 0.008 |  |  | <0.001 |  |  | <0.001 |  |  | 0.534 |  |  | 0.004 |
| - | 9803 | 58.08 | 41.92 |  | 83.29 | 16.71 |  | 89.46 | 10.54 |  | 89.95 | 10.05 |  | 89.89 | 10.11 |  |
| + | 183 | 48.09 | 51.91 |  | 70.49 | 29.51 |  | 79.78 | 20.22 |  | 88.52 | 11.48 |  | 83.06 | 16.94 |  |
| Iron |  |  |  | 0.434 |  |  | 0.236 |  |  | 0.006 |  |  | 0.923 |  |  | 0.064 |
| - | 9904 | 57.94 | 42.06 |  | 83.10 | 16.90 |  | 89.37 | 10.63 |  | 89.92 | 10.08 |  | 89.82 | 10.18 |  |
| + | 82 | 53.66 | 46.34 |  | 78.05 | 21.95 |  | 79.27 | 20.73 |  | 90.24 | 9.76 |  | 82.93 | 17.07 |  |
| Zinc |  |  |  | 0.299 |  |  | 0.091 |  |  | 0.024 |  |  | 0.774 |  |  | 0129 |
| - | 9909 | 57.95 | 42.07 |  | 83.16 | 16.89 |  | 89.40 | 10.65 |  | 89.96 | 10.09 |  | 89.85 | 10.20 |  |
| + | 77 | 51.95 | 48.05 |  | 75.32 | 24.68 |  | 80.52 | 19.48 |  | 90.91 | 9.09 |  | 84.42 | 15.58 |  |
| DHA |  |  |  | 0.053 |  |  | 0.018 |  |  | 0.042 |  |  | 0.111 |  |  | 0.258 |
| - | 9932 | 57.97 | 42.03 |  | 83.13 | 16.87 |  | 89.34 | 10.66 |  | 89.96 | 10.04 |  | 89.79 | 10.21 |  |
| + | 54 | 44.44 | 55.56 |  | 70.37 | 29.63 |  | 79.63 | 20.37 |  | 83.33 | 16.67 |  | 85.19 | 14.81 |  |

Note. Symbol “-” represented non-using nutrient supplements or without disease, and “+” represented the use of nutrient supplements or with disease. The figure below the symbol represented the proportion of older people using or non-using specific nutrient supplements. Chronic diseases were also described in this way. P-values were calculated using the chi-square.
